# Supplementary material for: Predictive nomogram for lymph node metastasis and survival in gastric cancer using contrast-enhanced computed tomography-based radiomics: a retrospective study
Source: PeerJ. 2024 Mar 20;12:e17111. doi: 10.7717/peerj.17111 (PMC10960528; doi:10.7717/peerj.17111)
Supplement: Supplemental Information 1 [file peerj-12-17111-s001.docx]

**Parameter adjustment and Z-score standardization**

The initial computed tomography image, along with the designated region of interest, were meticulously preserved as medical digital imaging files in the Nearly Raw Raster Data (NRRD) format. Subsequently, automated feature extraction was conducted employing Pyradiomics within the Python environment (version 3.7.2; accessible at https://python.org/). For feature extraction, the following parameters were fine-tuned: normalize, set to false; padDistance, set to 10; and Original^1^; Wavelet^1^; and the remaining parameters were left at their default settings. The feature extraction process encompassed a total of 18 first-order features (firstorder), 14 shape-related features (shape-3D), 22 features based on gray-level co-occurrence matrix, 22 features based on gray-level run length matrix, 16 features derived from gray-level size zone matrix, 5 features related to neighboring gray tone difference matrix, and 14 features based on gray-level dependence matrix. Following wavelet transform, the cumulative count of features reached a total of 833.^1^ All 833 features extracted from the dataset comprising 142 patients underwent a standardization process employing the Z-score method, whereby the mean and standard deviation of the training group were utilized as reference values. In essence, the Z-score normalization entails adjusting the data to adhere to a normal distribution, thereby ensuring that the processed data exhibits a mean of 0 and a standard deviation of 1. For the sequence$X=\left[ x_{1},x_{2},\ldots,x_{n} \right]$, the formula of the Z-score transform is as follows：$y_{i}=\frac{x_{i}-\bar{x}}{\sigma\left( X \right)}$ ，of which：$\bar{x}=\frac{1}{n}\sum_{i=1}^{n} x_{i}$，$\sigma\left( x \right)=\sqrt{\frac{n}{\left( n-1 \right)}\sum_{i=1}^{n} \left( x_{i}-\bar{x} \right)^{2}}$, and to finally obtain the standard sequence, $Y=\left[ y_{1},y_{2},\ldots,y_{n} \right]$

**Reference**

1. van Griethuysen JJM, Fedorov A, Parmar C, Hosny A, Aucoin N, Narayan V, et al. Computational radiomics system to decode the radiographic phenotype. Cancer Res. 2017;77: e104-7; doi: 10.1158/0008-5472.CAN-17-0339
